# Magnocellular visual function in developmental dyslexia: deficit in frequency-doubling perimetry and ocular motor skills

**DOI:** 10.5935/0004-2749.20210069

**Published:** 2021

**Authors:** Douglas de Araújo Vilhena, Márcia Reis Guimarães, Ricardo Queiroz Guimarães, Ângela Maria Vieira Pinheiro

**Affiliations:** 1 Laboratory of Applied Research in Neuroscience of Vision, Hospital de Olhos de Minas Gerais-Dr. Ricardo Guimarães, Belo Horizonte, MG, Brazil; 2 Laboratory of Cognitive Processes, Graduate Program on Psychology: Cognition and Behavior, Universidade Federal de Minas Gerais, Belo Horizonte, MG, Brazil; 3 Faculty of Psychology and Education Science, Universidade do Porto, Porto, Portugal

**Keywords:** Dyslexia, Reading, Visual perception, Vision disorders, Oculomotor muscles, Eye movements, Dislexia, Leitura, Percepção visual, Transtornos da visão, Músculos oculomotores, Movimentos oculares

## Abstract

**Purpose:**

This study aimed to verify if patients with developmental dyslexia present deficits coherent with visual magnocellular dysfunction.

**Methods:**

Participants with confirmed diagnosis of developmental dyslexia (*n*=62; age range=8-25 years; mean age=13.8 years, standard deviation=3.9; 77% male) were compared to a control group with normal development, matched for age, sex, ocular dominance, visual acuity, and text comprehension. The frequency-doubling technology perimetry was used to evaluate the peripheral visual field contrast sensitivity threshold. The Visagraph III Eye-Movement Recording System was used to evaluate ocular motor skills during text reading.

**Results:**

The developmental dyslexia group had significantly worse contrast sensitivity in the frequency-doubling technology, with strong effect size, than the matched control group. The developmental dyslexia group had more eyes classified in the impaired range of sensitivity threshold to detect frequency-doubling illusion than the control group. Moreover, the developmental dyslexia group had poorer ocular motor skills and reading performance, revealed by a difference in ocular fixations, regressions, span recognition, reading rate, and relative efficiency between groups. A significant correlation was found between contrast sensitivity and ocular motor skills. Participants with good relative efficiency had significantly better contrast sensitivity than participants with poor relative efficiency.

**Conclusions:**

The developmental dyslexia group presented a markedly worse performance in visual variables related to visual magnocellular function (*i.e.*, frequency-doubling technology perimetry and ocular motor skills) compared with a matched control group. Professionals need to be aware of the importance of evaluating vision of individuals with developmental dyslexia beyond visual acuity and including in their assessments instruments to evaluate temporal processing, with contrast sensitivity threshold.

## Abstract (pt)

**Objetivo:**

Verificar se pacientes com dislexia do de senvolvimento (DD) apresentam déficits coerentes com uma disfunção magnocelular visual.

**Métodos:**

Participantes com diagnóstico confirmado de dislexia do desenvolvimento (*n*=62; faixa etária=8 a 25 anos; Média da idade=13.8 anos, desvio padrão=3.9; 77% homens) foram comparados a um grupo controle com desenvolvimento típico, pareado por idade, sexo, dominância ocular, acuidade visual e compreensão de texto. A perimetria *Frequency-Doubling Technology* avaliou o limiar de sensibilidade ao contraste do campo visual periférico. O rastreador ocular Visagraph-III registrou os movimentos dos olhos durante leitura de texto.

**Resultados:**

O grupo com dislexia do desenvolvimento apresentou piores limiares de sensibilidade no *Frequency-Doubling Technology*, com tamanho de efeito forte, do que o grupo controle. O grupo com dislexia do desenvolvimento apresentou mais olhos classificados com déficits na sensibilidade à ilusão de frequência duplicada do que o grupo controle. O grupo com dislexia do desenvolvimento apresentou pior habilidade motora ocular e no desempenho de leitura, revelado pela diferença entre os grupos em relação às fixações oculares, regressões, alcance de reconhecimento, taxa de leitura e eficiência relativa. Foi encontrada correlação significativa entre a sensibilidade ao contraste e as habilidades motoras oculares. Os participantes com boa eficiência relativa apresentaram uma sensibilidade ao contraste significativamente melhor do que os participantes com baixa eficiência relativa.

**Conclusões:**

O grupo com dislexia do desenvolvimento apresentou desempenho inferior nas variáveis visuais relacionadas à função visual magnocelular (*i.e.*, perimetria de frequência duplicada e habilidades motoras oculares), quando comparado ao grupo controle pareado. Os profissionais precisam estar cientes da importância de investigar a visão dos pacientes com dislexia do desenvolvimento além da acuidade visual e incluir nos seus procedimentos diagnósticos instrumentos para avaliar o processamento temporal, com limiar de sensibilidade ao contraste.

## INTRODUCTION

Developmental dyslexia (DD) is a reading disorder that needs to be analyzed in a coherent framework perspective that includes the genetic level (e.g., incidence in the family), brain level (e.g., magnocellular and cerebellar deficit), cognitive level (e.g., deficits in phonology, processing speed, speech rhythm, visuospatial attention, sensory integration), and directly observable behavior level (e.g., reading, spelling, and writing)^(1)^.

Studies focusing on sensory integration demonstrated that children with neurodevelopmental disorders have fewer attentional resources available to correctly perform ocular motor tasks with high attentional load, thus exhibiting impairment in maintaining good level of postural stability, especially in a standing position compared with a sitting position^(2-5)^. Moreover, it was demonstrated that patients with DD showed deviant subjective visual vertical perception (*i.e.*, ability to estimate gravitational verticality in relation to the earth in the absence of any external reference frame) compared to controls^(6)^. Thus, the hypothesis underlying these somatosensorial studies is an impairment or immaturity in cerebellar integration of complex sensory inputs in neurodevelopmental disorders, with poor use of sensory information to compensate natural body perturbation.

Another parallel line of research at the brain level est ablished the hypothesis that DD present deficits in the magnocellular pathways and part of the posterior cortical attentional network involved in eye movement control^(7-10)^. The retinocortical/subcortical magnocellular visual pathway is mainly involved in temporal processing, object/word location (where), eye movement control, and attention control. These are essential cognitive features during reading activities, as the eyes have to systematically and sequentially make horizontal saccades (controlled by magnocellular pathway), followed by eye fixations of 200-400 ms (to extract and process the content via the parvocellular pathway), while coordinated binocular eye activity tracks line by line along a text. The magnocellular system plays a vital role in controlling visual attention to reading, which contributes to quick and precise recognition of each sequential letter within a word^(11)^.

Regarding measurable visual aspects, different studies demonstrated abnormal ocular motor skills in patients with DD compared to peers with normal development, such as frequent saccades of small amplitude, unstable fixation, higher number of unwanted saccades, high number ocular regressions while reading, atypical ocular tracking, less eye movement control in voluntary convergence, poor binocular coordination, and deficit in vergence movements^(7-8,12-14)^.

Besides ocular motor skills, DD may also be objectively identified by a deficit in motion perception^(15-17)^. In a seminal study^(15)^, 21 participants with DD presented worse performance on detection of the frequency-doubling illusion perimetry than 19 control normal readers, being less sensitive across the retina (p<0.005). A more recent study^(16)^ verified that a group of illiterate adults and normal and semi-illiterate readers performed specific spatial and temporal tasks related to visual magnocellular system, with all three groups performing better than the DD group (p<0.005). The authors^(16)^ concluded that this functional failure is probably not a consequence of a lack of reading skills and points to a causal role of magnocellular processing.

The present analytical study hypothesizes that participants with DD present deficits in the magnocellular system (peripheral vision and ocular motor skills), concomitant with a preserved parvocellular system (central vision acuity). This study aimed to verify if participants with DD, evaluated objectively by means of a frequency-doubling technology and eye tracker, present deficits coherent with a magnocellular dysfunction compared to a matched control group.

## METHODS

### Participants

This retrospective clinical controlled study was conducted in full accordance with the Declaration of Helsinki and approved by the ethics committee of the Universidade Federal de Minas Gerais. During the first meeting, all participants’ parents or legal guardians provided informed consent (and participants assented) to participate in a future study.

We reviewed consecutive case records of all patients who had been assessed from January 2007 to April 2018 at the NeuroVision Department of the Hospital de Olhos de Minas Gerais-Dr. Ricardo Guimarães. From this large data pool, we only selected the records of patients with a formal diagnosis of DD (DD group, *n*=62; mean age=13.8 years, standard deviation [SD]=3.9 years; age range: 8-25 years; 77% male; 37% left ocular dominance), based on professional assessments according to the Diagnostic and Statistical Manual of Mental Disorders 5th edition, who had good binocular visual acuity (better than 20/20 Snellen chart) and no comorbidity of other developmental disorders.

The control group consisted of typically developing participants, matched for age, sex, ocular dominance, and visual acuity (*n*=62; mean age=13.8 years, SD=4.4 years; age range: 8-25 years; 77% male; 37% left ocular dominance). All 124 participants were native Brazilian Portuguese speakers. The exclusion criteria from the data pool were as follows: (a) diagnosis of another developmental disorder (e.g., attention-deficit/hyperactivity disorder); (b) informal diagnosis of DD (*i.e.*, presumptive diagnoses made by parents, health professionals, or special needs’ teachers); (c) age >25 years; (d) poor visual acuity (worse than 20/30 Snellen chart); (e) color blindness (Pseudoisochromatic Ishihara 25 Plates Test and Farnsworth D15 Dichotomous Test); or (f) text comprehension <60% of correct answers.

### Instruments

The frequency-doubling technology (FDT, Humphrey Instruments) verifies the integrity of the peripheral visual field. FDT is used to analyze the contrast sensitivity, that is, the ability to recognize small differences in luminance or differentiate two objects from each other and the back ground. Each eye was measured separately at all 19 retinal regions using a full threshold analysis program (N-30). Each stimulus is formed by a low spatial frequency (vertical, cosinusoidal grid, 0.25 cycles per degree) and a high temporal frequency (flicker counter-phase of 25 Hz). The mean deviation (MD) index represents the average contrast sensitivity deviation from a normal person of the same age (based on normative database) and can either be a negative or positive value depending on the individual’s general contrast sensitivity, if it is below or above the average for that same age group. The pattern standard deviation (PSD) index reflects the roughness (focal-cluster alteration) of the visual field. The MD and PSD indices are reported in decibel (dB).

The Visagraph III Eye-Movement Recording System (Taylor Associates, New York) is used to verify the ocular motor skills and reading parameters. This system uses lens-free goggles with inbuilt infrared sensors to record eye movements during text reading. The binocular eye position (border between the iris and the sclera) is sampled with 60 Hz. The equipment’s algorithm only evaluates horizontal saccades and compensates for head movements. The following ocular motor and reading parameters were measured and analyzed: (a) ocular fixations, number of eye pauses (stationary periods) in reading from left to right per 100 words; (b) regressions, number of times eye movements are directed from right to left per 100 words; (c) span of recognition, number of words read divided by the number of fixations; (d) reading rate, number of words read in 1 min; (e) relative efficiency, reading rate divided by fixations and regressions; and (f) text comprehension, percentage of correct answers in a ten yes/no questionnaire concerning the content of the text that was read.

### Procedures

The FDT is used to verify the minimum contrast necessary to detect the stimulus, in each of the 19 locations, employing a modified binary search type of staircase strategy. If the stimulus is detected, the contrast is decreased in the following presentation; if the stimulus is not detected, the contrast is increased until the stimulus threshold with the lowest contrast is detected. The left eye was always tested first, followed by the right eye. The participant was instructed to look at the fixation point throughout the entire test and press the response button each time they saw a pattern.

Visagraph-III Eye-Tracking System was aligned to each participant’s interpupillary distance, considering any refractive corrections. All participants were provided with a text appropriate for their reading level and cognitive capacity to minimize abnormal reading eye movements and allow continuous reading performance to be recorded. Participants read the texts aloud from a viewing distance of 40-45 cm, in sitting position, and under standard office lighting (two-tube cool-white fluorescent lamp ceiling fixtures; 20-W 60-cm tubes; correlated color temperature, 5,000 K; 120 Hz flicker cycle). The reading material consisted of a single paragraph of black text, printed on a white paper, in Times New Roman font size 18. Data from the first and last lines were excluded from the analysis. After reading, participants answered ten questions about the text, with a comprehension score ≥60% qualified as typical reading performance.

### Data analysis

We used IBM SPSS Statistics (version 21.0, Chicago, IL) for all data analyses. Descriptive statistics included the mean and standard deviation. The best-corrected visual acuity values were converted to the logarithm of the minimal angle resolution scale. Statistical analysis was performed using independent Student’s *t*-test for the control variables, and an analysis of covariance (ANCOVA) (covariates, age and sex) for the FDT and Visagraph variables. Pearson bivariate correlations were used between FDT and Visagraph. Cohen’s *d* determined the clinical significance of group differences, with effect size interpreted using the criteria of 0.2 for a small effect, 0.5 for a medium effect, and 0.8 for a large effect. Chi-square (χ^2^) test was used to determine the significant differences between categorical data, with Phi (φ) used to indicate the strength of the relationship of 2 × 2 contingency tables. The significance level was set at <0.05.

## RESULTS

The DD and control group had no significant group difference in demographic variables (age range and mean, sex, ocular dominance) (p>0.05) (Table 1). The mean visual acuity (monocular and binocular) was also not significantly different between the two groups, corresponding to a 20/20 Snellen chart acuity. Likewise, the DD group (78%) and control group (81%) presented an equivalent performance in text comprehension, with no significant difference (p*=*0.51).

**Table 1:** Mean ± standard deviation of control variables (demographic and central visual function), peripheral visual field function (frequency-doubling technology), ocular motor skills, and reading parameters from the DD group and matched control group

Parameters	DD group	Control group	F	p	d
**Demographic and control variables** *Sample size (n)*	62	62		n/s	n/s
*Age range (years)*	8-25	8-25		n/s	n/s
*Mean age (M ± SD)*	13.8 ± 3.9	13.8 ± 4.4		n/s	n/s
*Male (%)*	77.4	77.4		n/s	n/s
*Left ocular dominance (%)*	37.1	37.1		n/s	n/s
*Monocular visual acuity (logMAR)*	0.02 ± 0.12	0.03 ± 0.12		n/s	n/s
*Binocular visual acuity (logMAR)*	-0.05 ± 0.11	-0.10 ± 0.10		n/s	n/s
*Text comprehension (%)*	78 ± 17	81 ± 16		n/s	n/s
**Peripheral visual field function (FDT)**					
MD both eyes (dB)^**^	-3.5 ± 3.4	-0.8 ± 1.8	70.0	<0.0001	0.99
MD left eye (dB)^**^	-3.4 ± 2.9	-0.9 ± 1.5	38.6	<0.0001	1.08
MD right eye (dB)^**^	-3.7 ± 3.7	-0.7 ± 2.1	32.9	<0.0001	1.00
MD dominant Eye (dB)^**^	-3.3 ± 3.0	-0.7 ± 1.8	36.1	<0.0001	1.50
MD nondominant Eye (dB)^**^	-3.8 ± 3.7	-0.8 ± 1.9	33.9	<0.0001	1.15
MD better eye (dB)^**^	-2.6 ± 2.8	0.1 ± 1.8	44.7	<0.0001	1.15
MD worse eye (dB)^**^	-4.5 ± 3.6	-1.6 ± 1.4	35.3	<0.0001	1.06
Eyes worse than MD -2.0 dB (%)^**^	65	26	-	<0.0001	φ0.48
PSD both eyes (dB)^**^	6.2 ± 2.8	5.2 ± 2.6	9.0	=0.0030	0.37
**Ocular motor skills and reading parameters (Visagraph-III)** Fixations^**^	196 ± 130	155 ± 84	8.5	=0.0038	0.37
Regressions^*^	61 ± 63	45 ± 34	5.8	=0.0164	0.32
Span of recognition (%)^**^	65 ± 31	87 ± 63	11.6	=0.0008	0.44
Reading rate (words per minute)^**^	158 ± 92	212 ± 124	9.7	=0.0023	0.49
Relative efficiency^**^	1.1 ± 1.2	2.2 ± 4.9	13.8	=0.0003	0.31

FDT=frequency-doubling technology; MD=mean deviation index; PSD=pattern standard deviation; dB=decibel;

* =Significance level at 0.05;

** =Significance level at 0.01; n/s=not significant; DD=developmental dyslexia.

**Figure 1:**
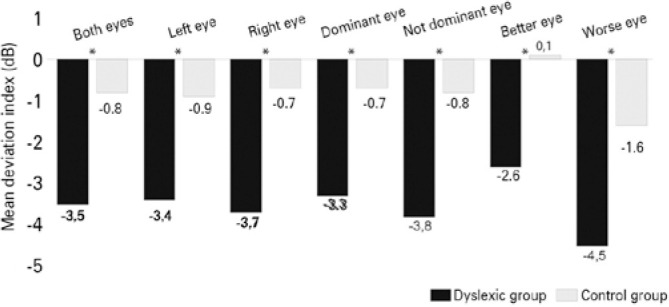
Frequency-doubling technology mean deviation index (dB) between the developmental dyslexia group and control group. *Significance level at 0.001.

The FDT MD index averaged over the two eyes (MD both eyes) for the DD group was *M*=-3.5 dB, significantly worse than *M*=-0.8 dB of the control group [*F*_(3,245)_=70.0, p<0.0001, *d*=0.99] (Table 1 and Figure 1). This pattern of significantly worse performance of the DD group in the FDT MD index (p<0.0001), with strong effect size compared to the control group, occu rred even in the analyses of the eyes: (a) left side [*F*_(3,121)_=38.6, *d*=1.08], (b) right side [*F*_(3,121)_=32.9, *d*=1.00], (c) dominant [*F*_(3,121)_=36.1, *d*=1.50], (d) not dominant [*F*_(3,121)_=33.9, *d*=1.15], (e) better performance [*F*_(3,121)_=44.7, *d*=1.15], and (f) worse performance [*F*_(3,121)_=35.3, *d*=1.06] (Table 1 and Figure 1).

Overall, 65% of the eyes in the DD group had an FDT MD index in the impaired range of sensitivity (classified as a visual contrast threshold worse than -2.0 dB). A proportion of participants had significantly higher index than 26% of the control group (difference=39%; χ^2^=30.7; p<0.0001; φ=0.48). The FDT PSD index averaged over the two eyes for the DD group was *M*=6.2 dB, significantly worse than *M*=5.2 dB of the gcontrol group [*F*_(3,121)_=9.0, p*=*0.0030, *d*=0.37] (Table 1).

For the ocular motor skills, ANCOVA revealed a statistically significant difference in fixations [*F*_(3,121)_=8.5; p=0.0038, *d*=0.37], regressions [*F*_(3,121)_=5.8; p*=*0.016, *d*=0.32], span recognition [*F*_(3,121)_=11.6; p*=*0.0008, *d*=0.44], reading rate [*F*_(3,121)_=9.7; p*=*0.0023, *d*=0.49], and relative efficiency [*F*_(3,121)_=13.8; p*=*0.0003, *d*=0.31] between the DD and control groups, with a small effect size (Table 1). *Post hoc* analysis showed that the DD group had a poorer ocular motor skill and reading performance than the control group.

Pearson’s bivariate analysis showed a correlation between FDT MD index average of both eyes and fixations (r=-0.15, p*=*0.02), span recognition (r=0.17, p*=*0.016), reading rate (r=0.24, p*=*0.0002), and relative efficiency (r=0.18, p*=*0.006), with the exception of regression (p*=*0.11). Participants with good relative efficiency (*n*=28 participants with score of ≥2.0) had an FDT MD index significantly better than participants with poor relative efficiency (*n*=61 participants with score of ≤0.9) [*M*=-1.2 dB *vs* -2.7 dB, *F*_(1,88)_=7.1, p*=*0.008, *d*=0.45].

## DISCUSSION

This study aimed to verify if participants with DD, evaluated using a FDT and eye tracker, present deficits in magnocellular visual function parameters compared to a matched control group for sample size, age, sex, ocular dominance, visual acuity, and text comprehension. The equivalence between groups ensured comparable data and increased the reliability of the eye-tracking data recorded, with participants reading to comprehend the content of the text.

The FDT was developed based on particular neural magnocellular characteristics and can be used to examine the magnocellular dysfunction hypothesis in DD. FDT provides a MD index to generally summarize the visual field contrast sensitivity threshold. For the peripheral visual function, the DD group had a decreased sensitivity on the detection of the FDT illusion than the control group, even if we divided the data by sides (left and right), ocular dominance (dominant and nondominant), or performance (better and worse) (p<0.0001). The FDT MD sensitivity for the eye with worse performance in the current study (DD=-4,5 dB vs. control=-1.6 dB) was similar to the reference study^(15)^ (DD=-5,01 dB vs control=-0,46 dB). The PSD index was also significantly different between the DD and control groups.

The deficit in the detection of the frequency-doubling perimetry illusion indicates a visual magnocellular dysfunction in the DD group, which can explain the poorer ocular motor skill, compared to the control group. Eye movement recorded while text reading (Visagraph-III) demonstrated that the DD group had a significantly higher number of ocular fixations and regressions, narrower span of recognition (amount of information perceived in each eye fixation), slower reading rate, and poorer relative efficiency than the control group (p<0.05), while maintaining an equal text comprehension.

One novelty of the current study is the group difference in FDT’s MD between participants with good and poor reading efficiency. A significant correlation, although weak, was found between FDT MD index and ocular motor reading parameters of fixations, span recognition, reading rate, and relative efficiency. These participants with good relative efficiency (parameter that combines fixations, regressions, and reading rate) had an FDT MD index significantly better than participants with poor relative efficiency (p*=*0.008). These are coherent with the reference study^(15)^ that demonstrated a significant correlation between FDT MD and reading lag (number of years deviation between chronological age and reading age) (r=-0.57, p<0.01), with children who have a higher reading lag also are proportionally less sensitive to the spatial frequency-doubling illusion.

Moreover, 74% (3:1) of the current sample are male, coherent with sex bias toward men for the incidence of reading disabilities. It is estimated that boys are 2:1 to 5:1 more likely to be identified as having DD than girls^(18-20)^. A study with magnetic resonance imaging^(21)^ verified neuroanatomical sex differences in DD, with less gray matter volume identified in men with DD (left middle/inferior temporal gyri and right postcentral/ supramarginal gyri), boys with DD (left supramarginal/ angular gyri), woman with DD (right precuneus and paracentral lobule/medial frontal gyrus), and girls with DD (right central sulcus, adjacent gyri, left cuneus) compared to controls without DD. The authors^(21)^ argued that women have less involvement of left hemisphere language regions but rather early sensory and motor cortices (*i.e.*, motor and premotor cortex, primary visual cortex). In the current study, the demographic matched control group and the ANCOVA analysis confirmed that age and sex did not explain the group differences in FDT and Visagraph.

To the best of our knowledge, this is the first study on FDT that evaluated a sample of DD participants with Portuguese as their native language. One strength of the current study is the sample size, larger than those in the reference studies^(7,15,16)^. Another strength is the homogeneity of the sample, as only individuals with formal diagnosis of DD were selected, together with the exclusion of 57 individuals with DD from the data pool due to comorbidity with attention-deficit/hyperactivity disorder. The DD group presented a markedly worse performance in visual variables related to magnocellular visual function (*i.e.*, peripheral visual function and ocular motor skills) compared to a matched control group. Thus, we could objectively identify physical evidence of visual-related reading difficulties, such as poorer contrast sensitivity thresholds and higher number of ocular fixations and regressions.

A dysfunctional visual magnocellular system may be in the core of some individuals with DD, having a causal relationship to reading difficulty^(8,17)^. A dysfunctional magnocellular system induces visual stress conditions that hinder the development of a proficient, comfortable, and sustained reading. Over a sustained reading of a book, for example, the accumulated visual activity can lead to visual stress symptoms, such as poor ocular motor skills, visual distortions, reading difficulties, and discomfort, frequently reported by patients with DD and poor readers^(8-11,22)^.

The results of this study demonstrate the importance for ophthalmology clinics to evaluate individuals with DD beyond visual acuity and include in their assessments instruments to evaluate ocular motor skills and visual temporal processing, with contrast thresholds. The parvocellular and magnocellular visual pathways are directly involved in proficient reading, as they are parallel and partially dependent systems. Although it is intuitive to think that foveal vision (parvocellular visual pathway) is important to extract high-resolution spatial information of letters and words, the parafoveal region (mainly magnocellular visual pathway) is fundamental during proficient reading to predirect the ensuing saccade to the next optimal fixation point and allow fluent reading^(23,24)^.

Therefore, understanding the dynamics of visual information processing during reading is important as it (a) reveals trends and existing gaps in the field, (b) guides the development of future studies, and (c) maximizes investments to increase knowledge. The current findings improve our understanding on the mechanism underlying the visual function in DD and may prompt advances in strategies to prevent the onset and progression of reading difficulties. Although visual acuity is fundamental in extracting static information of letters and words, proficient reading involves a dynamic visual activity with temporal sequence processing of visual information to form precise representations of the visual sequencing of letters^(11)^. It is imperative to facilitate the development of a simple and powerful diagnostic tool for the evaluation and identification of DD and reading difficulties and of an efficient therapeutic strategy to help practitioners with clinical decision-making.
